# Nationwide Seroprevalence Survey of *Angiostrongylus vasorum-*Derived Antigens and Specific Antibodies in Dogs from Colombia

**DOI:** 10.3390/microorganisms10081565

**Published:** 2022-08-04

**Authors:** Manuel Uribe, Lisa Segeritz, Manuela Schnyder, Anja Taubert, Carlos Hermosilla, Sara López-Osorio, Agustín Góngora-Orjuela, Jenny J. Chaparro-Gutiérrez

**Affiliations:** 1 Biomedical Research Center Seltersberg (BFS), Institute of Parasitology, Justus Liebig University Giessen, 35392 Gießen, Germany; 2 CIBAV Research Group, Veterinary Medicine School, Universidad de Antioquia, Medellín 050034, Colombia; 3 Institute of Parasitology, Vetsuisse Faculty, University of Zurich, Winterthurerstrasse 266a, 8057 Zurich, Switzerland; 4 Facultad Ciencias Agropecuarias y Recursos Naturales, Universidad de Los Llanos, Villavicencio 500017, Colombia

**Keywords:** *Angiostrongylus vasorum*, ELISA, canine angiostrongylosis, epidemiology, South America

## Abstract

*Angiostrongylus vasorum* is a cardiopulmonary nematode, causing several clinical manifestations in dogs, e.g., severe respiratory signs, coagulopathy, and gastrointestinal or neurological signs. In the last decades, this parasite has been described to spread and emerge in Europe and North America. Scant studies on *A. vasorum* occurrence in South America exist. Recently, *A. vasorum* was detected in gastropod intermediate hosts in Colombia, where data on definitive host prevalence, e.g., dogs and wild canids, are still limited. Therefore, the sera of 955 dogs, varying in age and breed from seven different departments all over Colombia, were collected and analysed for *A. vasorum* antigens and parasite-specific antibodies by ELISA. In total, 1.05 % (*n* = 10; 95 % CI 0.40–1.69) of the samples were antigen-positive and 2.62 % (*n* = 25; 95 % CI 1.61–3.63) were antibody-positive. These results confirm the presence of *A. vasorum* in Colombia, although positive results in antigen and antibody reactions in the same dog were not detected. This study is the first large-scale survey on *A. vasorum* seroprevalences in dogs from Colombia.

## 1. Introduction

The canid heartworm *Angiostrongylus vasorum* is a metastrongyloid lungworm species belonging to the most pathogenic cardiorespiratory parasites in dogs, and also infects a broad range of wild carnivore families, e.g., Canidae, Mephitidae, Mustelidae, Procyonidae, and Ailuridae [1,2]. This nematode has a heteroxenous lifecycle, where terrestrial gastropods act as natural obligate intermediate hosts [3]. Clinical manifestations of angiostrongylosis in dogs vary broadly from non-specific signs like lethargy, anorexia, diarrhoea, and weight loss to more specific signs such as coughing, dyspnoea, and haemorrhages caused by verminous pneumonia and disseminated coagulopathies [4,5,6]. Furthermore, canine angiostrongylosis can be associated with neurological signs, ocular manifestations, or even pneumothorax due to alveolar rupture [7,8,9,10,11]. Domestic dogs may be clinically unapparent for months to years before manifesting clinical signs, or may become chronically ill, or even die acutely [12]. Because subclinical *A. vasorum* infections can occur, detecting infected dogs can be difficult. Thus, veterinarians’ awareness of this parasitic disease is important for the early detection of the infection, thereby performing an appropriate diagnostic interpretation, anthelmintic treatments, and preventive measures. Rapid serological assays, ultrasonography, nonclassical coprological tests such as Mini-FLOTAC, and molecular detection, but also classical and low-cost Baermann funnel techniques, have all shown usefulness in *A. vasorum* diagnosis [13]. A rapid and precise diagnosis is essential, not only for proper anthelminthic treatments, but also for disease control among host populations [14,15,16]. The advantages of serological assays like ELISAs principally are the possibility to detect non-patent infections and to test efficiently large groups of animals, and to therefore better understand epizootiology of canine angiostrongylosis [17].

**Figure 1:**
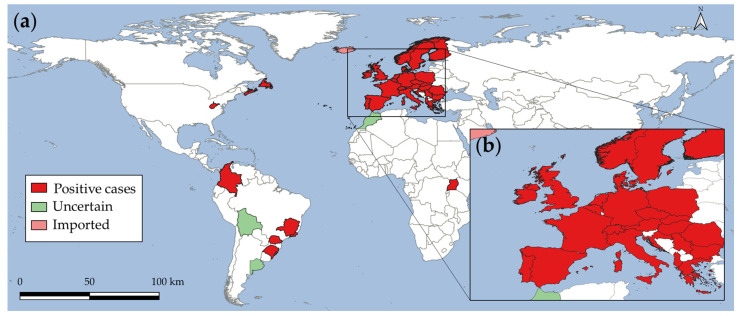
Worldwide geographical distribution range of *Angiostrongylus vasorum*. (**a**) Map shows the global location of parasite case reports in definitive and intermediate hosts. (**b**) Europe close-up.

In the last decades, *A. vasorum* was reported to spread and emerge in previously supposed non-endemic regions [18,19,20]. The worldwide geographical distribution reveals the parasite’s occurrence in Africa, America, and Europe, which is shown in Figure 1 [2,19,21,22,23,24,25]. To the best of our knowledge, to date there have been no autochthonous occurrence reports of this cardio–pneumotropic parasite in Asia, Oceania, or the Middle East, either in carnivore definitive hosts or in obligate gastropod intermediate hosts (e.g., snails, semi-slugs, and slugs). Scientific attention on this parasite was raised and many epidemiological studies, especially in Europe, were performed [2]. A wide distribution of the parasite is reported in this continent, despite the absence of reports for some European countries such as Andorra, Bosnia and Herzegovina, Kosovo, Liechtenstein, Luxembourg, and Slovenia. Notwithstanding, given the intermediate host density, spatial variations, and environmental factors that influence parasite transmission and local movement [26], it is very likely that the parasite occurs in these territories, but remains unreported for domestic dogs until now.

In Africa, the parasite was observed during the 1960s in Ugandan domestic dogs [22]. Additionally, in Morocco, a potential autochthonous *A. vasorum* case in a 6-month-old asymptomatic dog was reported [21]. For the North American subcontinent, there are *A. vasorum* reports in canids from West Virginia (USA), and Nova Scotia and Prince Edward Island in Canada [12,27]. The historically endemic focus for *A. vasorum* in North America occurs in the south-eastern part of Newfoundland island [12]. In South America, data on *A. vasorum* prevalence are still scarce and ambiguous ‘lungworm larvae’ descriptions make it difficult to establish the real parasite distribution in the Bolivian Chaco and Buenos Aires province in Argentina [19,28]. Throughout southern Brazil, natural *A*. *vasorum* infections were reported both in wild canids (i.e., *Cerdocyion thous* and *Lycalope*x *vetulus*) and domestic dogs in the state of Minas Gerais, Paraná, Rio de Janeiro, and Rio Grande do Sul [19,29]. Recently, *A. vasorum* larvae were found in the invasive giant African snail species *Lissachatina fulica* as well as other metastrongyloid lungworm species (i.e., *Troglostrongylus brevior*, *Crenosoma vulpis*, *Aelurostrongylus abstrusus*) in Colombia for the first time [30]. In definitive hosts, *A. vasorum* was identified in Colombia initially in a domestic dog sixty years ago by Gonçalves et al. (1961) in accordance with [31], and in 2014, another patent *A. vasorum* infection was reported in a crab-eating fox (*C*. *thous*), thus constituting the only two nationally published records of this metastrongyloid parasite [32]. Consistently, data on the actual prevalence or the spread of canine angiostrongylosis to new Colombian areas are still lacking, despite the overlapping spectrum of natural intermediate hosts and definitive hosts carrying *A. vasorum* infections [28,29]. Moreover, classical diagnostic methods such as the Baermann funnel technique, by which first-stage larvae (L1) can be microscopically identified in the faeces of definitive hosts, are rarely used in veterinary clinics around the country. Naturally *A*. *vasorum*-infected dogs may show highly variable clinical manifestations, thus the accurate, prompt, and effective diagnosis of this parasite is not a straightforward task [6,33]. As canine angiostrongylosis is a neglected disease and the knowledge of *A. vasorum* epidemiology in South America is still poorly understood, it can be easily overlooked by veterinary clinicians [19].

In previously published large-scale epidemiological studies on *A. vasorum*-infected dogs and foxes within Europe [34], novel diagnostic tools such as serology have frequently been used to reveal occurrences. Thus, the aim of the current epidemiological survey was to perform a nationwide prevalence survey for *A. vasorum* in Colombian domestic dogs using the combined detection of circulating antigens and specific antibodies through a serological approach.

## 2. Materials and Methods

Briefly, from March 2017 to May 2017, a total of 1024 domestic dog blood samples were collected from across Colombia through collaboration with veterinary clinicians, universities, veterinary diagnostic laboratories, and animal hospitals. Based on the Köppen-Geiger classification, the serological survey was carried out in Amazonian, Andean, Caribbean, Orinoco, and Pacific eco-epidemiological regions of Colombia, and in seven departments (i.e., Antioquia, Atlántico, Córdoba, Cundinamarca, Risaralda, Sucre, and Meta) throughout tropical rainforest (Af), tropical monsoon (Am), tropical wet savannah (Aw), and temperate oceanic (Cfb) climates [35].

The study inclusion criteria for dogs were being older than one month and not having received prophylactic anti-parasitic treatments. All sampled animals were owned pets or dogs from animal shelters; no stray or feral dogs were included. Whole blood (WB) samples were collected mainly from the venipuncture of the cephalic or jugular veins and placed into 3 mL vacutainer tubes without anticoagulant. The WB samples were stored at 4 °C for a maximum of 24 h before sera isolation, avoiding thawing and freezing the sample several times. Thereafter, the samples were centrifuged at 2200 rpm for 20 min to separate the sera and blood cells. The serum samples were stored at −20 °C until further use. From the total of the collected WB samples (*n* = 1024), a total of 979 serum samples were successfully obtained, and 963 samples were finally suitable for serological analyses, discarding haemolytic or lipaemic samples. Finally, a overall 955 sera samples were analysed, due to insufficient sample quantity and haemolysis of some samples. The sera (*n* = 955) were tested for the presence of circulating *A. vasorum* antigens using monoclonal and polyclonal antibodies in a sandwich ELISA, with a sensitivity of 95.7% and a specificity of 94.0%, as previously described [36], and for antibodies against *A. vasorum* using adult *A. vasorum* somatic antigen purified with monoclonal antibodies (mAb 5/5), with a sensitivity of 81% and a specificity of 98.8% [17]. Both tests were performed at the Institute of Parasitology, Vetsuisse Faculty, University of Zurich, Switzerland. Test thresholds were determined with 300 randomly selected samples based on the mean value of optical density (A_405_ nm) plus three standard deviations. All test runs included a background control, a conjugate control, three positive control sera from three experimentally infected dogs, and two control sera from negative dogs. For a complete summary list of each sample in which the sex, age, breed, origin, and serological results (Ag/Ab) are described, please refer to Table S1.

## 3. Results

**Figure 2:**
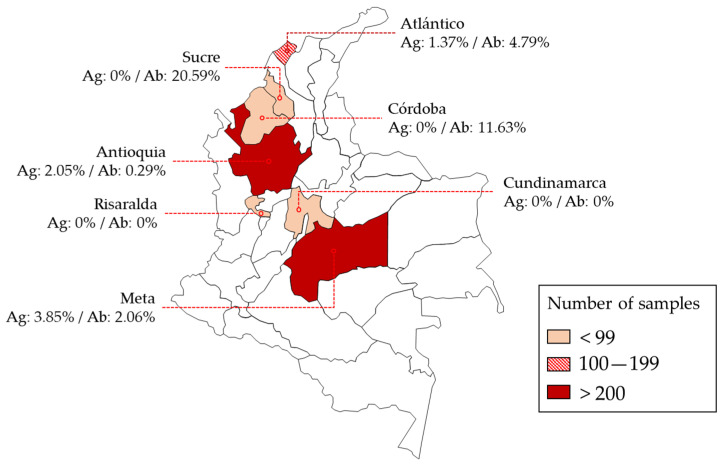
Geographical origins of domestic dogs (*n* = 955) serologically examined for *Angiostrongylus vasorum* occurrence. Different red shades represent the sampling density by area. Ag: antigen prevalence; Ab: antibody prevalence.

The *A. vasorum* antigen- and antibody-positive tested samples originated from different areas of Colombia, distributed in five out of seven departments (i.e., departments of Antioquia, Atlántico, Córdoba, Meta, and Sucre). The location and seropositivity of all tested samples is shown in Figure 2. 

Overall, 1.05% (*n* = 10; 95% CI 0.56–1.92) of the evaluated dog sera tested *A. vasorum* antigen-positive. Furthermore, 2.62% (*n* = 25; 95% CI 1.77–3.83) of the sampled dogs tested *A. vasorum* antibody-seropositive. Table 1 summarizes the seropositivity status of all examined animals. None of the analysed sera tested positive for both, i.e., *A. vasorum* antigen and antibody detection. Antigen-positive dogs originated from the municipalities of Medellín, Barranquilla, and Villavicencio. Antibody-positive dogs came, likewise, from the same areas with additional seropositive dogs coming from Montería, Sincelejo, and Cumaral. The origin of 43 samples was unknown. The prevalence of both antibodies and antigens was only reported in two municipalities (i.e., Barranquilla and Medellín). The highest regional antigen serological prevalence of *A. vasorum* was detected in Villavicencio at 3.85% (1 out of 26; 95% CI 0.68–18.89), followed by Medellín at 2.05% (7 out of 341; 95% CI 0.997–4.17), and Barranquilla at 1.37% (2 out of 146; 95% CI 0.37–4.85). On the other hand, major antibody reactivity was evidenced in Sincelejo at 20.59% (7 out of 34; 95% CI 10.34–36.79) and in Montería at 11.63% (5 out of 43; 95 % CI 5.07–24.47), both located in the north-western Caribbean region, characterized by a tropical wet savannah (Aw) climate. 

## 4. Discussion

Canine angiostrongylosis is an emerging and underestimated cardiopulmonary disease reported only sporadically in South America [37]. In contrast, serological studies carried out in European canids have shown an endemic occurrence and a widespread distribution of *A. vasorum* [34,38,39,40,41,42]. The current study presents first-time evidence of *A. vasorum* occurrence among a representative dog population (*n* = 955) in different Colombian regions, and thereby contributes to a deeper understanding of the actual prevalence in South American dogs. On this continent, the presence of “lungworm larvae” has been previously reported both in wild and domestic canids [28,43,44,45,46,47]. However, the identification of these larvae was not properly confirmed due to confusing morphological traits or non-specific serological reactivity. Notwithstanding*,* even new approaches to studying the morphological details of intra-molluscan stages of *A. vasorum* have been developed [48,49,50]. A prevalence of 3.9% *A*. *vasorum*-infected African giant snails (*Lissachatina fulica*; *n* = 609) was found in a preliminary gastropod survey in Colombia, which also reported the presence of the *A. vasorum* European genotype in South America [37]. The current study indicates that dogs act as definitive hosts in several regions of Colombia.

With an overall sera prevalence of 1.05%/2.62% (antigen/antibody), these results show similarities to European serological studies for *A. vasorum* infections, but the prevalence remains lower than the reported occurrence in Colombian gastropods [34,39,40,41,42]. While most of the *A. vasorum* prevalence data in definitive host populations is higher than the one of intermediate host populations, this discrepancy seems to be unexpected. As *A. vasorum* occurs in hyperendemic foci, the variable sampling locations selected in these different studies might be a possible explanation for the differences [51,52]. 

Here, we did not find any correlation between the detection of seropositivity for *A. vasorum* and the dog’s sex. Regarding geographical origins, in Sincelejo and Barranquilla, seven dogs were antibody-positive, respectively, followed by Montería and Cumaral, both with five positive animals, and one positive sample for Medellín. Additionally, 7 out of 10 antigen-positive samples came from the municipality of Medellín, Antioquia. 

It should be highlighted that wild definitive hosts, e.g., red foxes (*Vulpes vulpes*) in Europe, are reported to act as pivotal wild reservoir hosts, which additionally show higher prevalence than domestic dogs, and therefore play an important role in the spread of *A. vasorum* [53]. Hence, the current data might indicate that other wild canid hosts for *A. vasorum* in Colombia might also represent the natural reservoirs, having higher prevalence, as reported for red foxes in Europe. It is thus important to evaluate the *A. vasorum* infections among wild carnivores in non-endemic and unreported areas, as potential reservoir hosts for domestic animal infections [54]. Consequently, wild carnivores, distributed in the Neotropical realm, should be sampled in the future for a better understanding of *A. vasorum* epidemiology in this highly biodiverse region. Within the little existing data, *A. vasorum-*infected crab-eating foxes (*Cerdocyon thous*) have been reported and seem to be a potential definitive host. However, since the short-eared dog (*Atelocynus microtis*), maned wolf (*Chrysocyon brachyurus*), bush dog (*Speothos venaticus*), hoary fox (*Lycalopex vetulus*), sechuran fox (*Lycalopex sechura*), pampa’s fox (*Lycalopex gymnocercus*), Darwin’s fox (*Lycalopex fulvipes*), culpeo (*Lycalopex culpaeus*), chilla (*Lycalopex griseus*), and members of the superfamily Musteloidea, like tyra (*Eira barbara*), neotropical otter (*Lontra longicaudis*), ring-tailed coati (*Nasua nasua*), kinkajou (*Potos flavus*), and olingo (*Bassaricyon* sp.) could also be potential hosts for *A. vasorum*, further investigations are required to understand and establish the role of these species in the lifecycle, distribution, and epidemiology of the parasite in South and Central America.

The highest antibody-seroprevalence in the current study was calculated for Sincelejo/Sucre at 20.59% (95% CI: 10.35–36.80) and may indicate a hyperendemic focus area. Surprisingly, the antigen testing resulted negative in samples originating from this region. However, no sampling location was found to be positive for both antibody and antigen tests. A similar unexpected observation was found in other serological studies from dogs in Romania and Bulgaria [42,55]. Seroconversion is directly associated with the sampling time point. Antigen detection is possible 7–11 weeks after infection, while antibody seroconversion may occur as early as 3–6 weeks after infection. In contrast, after parasite elimination, antibodies may persist up to 9 weeks, while circulating antigens were not anymore detectable after 6 weeks. This may explain the generally observed higher number of antibody positive dogs compared to antigen positive dogs in this study [13].

To conclude, the current study confirmed the endemic occurrence of the cardiopulmonary nematode *A. vasorum* in Colombia. It also aims to raise the awareness of veterinary health staff of this neglected parasite in South America, with the goal of early diagnosing and performing treatment of affected dogs. Moreover, it calls for further research activities to evaluate the parasite occurrence among domestic dogs, other endemic wild canids (mainly foxes, bush dogs, and mane wolves), obligate gastropod intermediate hosts, and potential paratenic host populations within non-endemic regions in the Americas.

## Figures and Tables

**Table 1:** Serological results of dog sera samples from Colombia, tested for the presence of *A. vasorum* antigens and for specific antibodies against *A. vasorum*.

Departments	Municipalities	*n*	Antigen +	%	CI ^1^	Antibody +	%	CI ^1^
Antioquia	Bello	6	0	0.00%		0	0.00%	
	Caldas	1	0	0.00%		0	0.00%	
	Copacabana	5	0	0.00%		0	0.00%	
	El Retiro	1	0	0.00%		0	0.00%	
	Itagüi	5	0	0.00%		0	0.00%	
	La Ceja	3	0	0.00%		0	0.00%	
	Medellín	341	7	2.05%	0.99–4.17	1	0.29%	0.05–1.64
	Rionegro	3	0	0.00%		0	0.00%	
	Sabaneta	4	0	0.00%		0	0.00%	
Atlántico	Barranquilla	146	2	1.37%	0.37–4.85	7	4.79%	2.34–9.56
	Puerto Colombia	20	0	0.00%		0	0.00%	
Córdoba	Montería	43	0	0.00%		5	11.63%	5.07–24.47
Cundinamarca	NR **^2^**	39	0	0.00%		0	0.00%	
Risaralda	Pereira	31	0	0.00%		0	0.00%	
Sucre	Sincelejo	34	0	0.00%		7	20.59%	10.34–36.79
Meta	Cumaral	243	0	0.00%		5	2.06%	0.88–4.72
	Villavicencio	26	1	3.85%	0.68–18.89	0	0.00%	
	NR **^2^**	4	0	0		0	0	
	Total	955	10	1.05%	0.569–1.916	25	2.62%	1.77–3.83

^1^ CI = 95% confidence interval; **^2^** Not recorded. + positive serological reactivity.

## Data Availability

Not applicable.

## Supplementary Materials

The following supporting information can be downloaded at: https://www.mdpi.com/article/10.3390/microorganisms10081565/s1, Table S1: Dog serum database.
